# A COVID-19 Pivot Plan to Resume Elective Surgeries at the Hartford Healthcare Connecticut Orthopaedic Institute

**DOI:** 10.7759/cureus.15077

**Published:** 2021-05-17

**Authors:** Swaroopa Vaidya, Daniel Berluti, John F Irving, Gerard Girasole, John D McCallum, Leonard Kolstad, Tara McLaughlin

**Affiliations:** 1 Connecticut Orthopaedic Institute, St. Vincent's Medical Center, Hartford Healthcare, Bridgeport, USA; 2 Connecticut Orthopaedic Institute, MidState Medical Center, Hartford Healthcare, Meriden, USA; 3 Department of Research Administration, Hartford Healthcare, Hartford, USA

**Keywords:** total joint arthroplasty (tja), elective surgeries, pandemic, covid-19

## Abstract

Background

The Connecticut Orthopaedic Institute (COI) conceptualized a Pivot Plan during an elective surgery moratorium at the beginning of the severe acute respiratory syndrome coronavirus 2 (SARS-CoV-2) pandemic with the goal of planning and executing orthopedic procedures safely. With the resumption of elective surgeries and the continued planning of surgical recovery over the months (and possibly years) to follow, facilities must brace themselves for repeat waves of COVID-19. Thereby, herein we share the Pivot Plan, its implementation process, evaluation of patient safety, and program performance during a pandemic. This could inform the efforts of other institutions seeking to restart non-emergent surgeries during similarly trying times in the future.

Methods

The COI formed a multidisciplinary team of leaders that met weekly to design a Pivot Plan and a dashboard to guide the resumption of surgeries and assess the performance of the Pivot Plan. The plan revolved around four domains: safety, space, staff, and supplies. It was implemented in two COI-affiliated facilities: MidState Medical Center (MMC) and St. Vincent’s Medical Center (SVMC). Monthly metrics from May to November 2020 were compared to the six-month averages for the pre-pandemic baseline period from September 2019 to February 2020.

Results

The total number (N) of elective orthopaedic cases prior to the pandemic pre-COVID averaged 372 cases per month for MMC and 197 cases for SVMC. During the pandemic post-COVID, N averaging at 361 for MMC and 243 for SVMC illustrates COI was able to perform elective surgeries amid a worsening pandemic. Same-day (SD) discharge rates for total joint arthroplasty (TJA) pre-COVID averaged 8% for MMC and 3% for SVMC. Post-COVID, the SD average was 16.7% for MMC and 11.4% for SVMC. This data indicates that orthopaedic providers were cognizant of length of stay in order to reduce the risk of in-hospital exposure to COVID-19. The 30-day readmission (30R) rate for TJA pre-COVID averaged 1.4% for MMC and 2.7% for SVMC. A high level of care and follow-up is reflected in a lower average 30R post-COVID, 1.1% for both MMC and SVMC. Transitions for TJA patients to their home settings after surgery also reflect the quality of care and the efficiency of the patient throughput process with necessary precautions in place. Post-COVID, the patient transition to home (T) averaged 98.1% for MMC and 97.5% for SVMC compared to T = 96.8% for MMC and 88% for SVMC pre-COVID. No patients experienced deep vein thrombosis or pulmonary embolism during the time period of the project. Positive COVID-19 diagnosis 23 days after discharge was 0% at MMC and 0.2% at SVMC.

Conclusion

The COI Pivot Plan was successfully implemented at two different hospitals offering elective orthopaedic surgeries to a varied patient population. The precautions taken by COI were effective in controlling the spread of the SARS-CoV-2 virus while returning to elective orthopaedic surgery. Furthermore, data collected before and after the onset of the COVID-19 pandemic indicated that program performance and quality improved.

## Introduction

In the year 2020, the medical field was struck by an unprecedented pandemic of catastrophic proportions. According to the CDC, 2,074,529 cases of coronavirus 2019 (COVID-19) had been confirmed globally as of April 17, 2020 [1]. As COVID-19 spread throughout the United States (US), federal and state mandates forced most institutions to postpone elective surgical cases, creating a massive loss of revenue for healthcare systems [2]. Predictive modeling estimated that over 28 million operations could be canceled or postponed globally during the peak of the pandemic [3]. These necessary measures, taken at an extraordinary time, saved resources (including hospital beds and personal protective equipment) and helped to mitigate the spread of a highly contagious virus to surgical staff [4]. With the resumption of elective surgeries and the continued planning of surgical recovery over the months (and possibly years) to follow, facilities braced themselves for repeat waves of COVID-19 [3].

Due to the large number of unknowns surrounding the current pandemic, sharing strategies to safely resume surgery is crucial as healthcare systems seek to return to and maintain surgical volume during and after COVID-19. In this report, we describe the precautionary planning steps taken by the Connecticut Orthopaedic Institute (COI) and its parent corporation Hartford HealthCare (HHC) to resume elective orthopaedic surgeries after they were suspended due to COVID-19 and we share information regarding process changes and performance metrics. The COI holds The Joint Commission’s Advanced Certification for Total Hip and Knee Replacement surgery and performs over 2,000 total joint replacement surgeries annually. In response to operational changes that were required during the early stages of the pandemic, the COI formed a multidisciplinary team of leaders from programming, operations, marketing, and research that met weekly to design a Pivot Plan dashboard. The dashboard was based on countermeasures put into place as elective surgeries were resumed after the first COVID-19 peak in April 2020. Outcome metrics were tracked weekly on the dashboard. This “Pivot Plan” was implemented in two COI- affiliated facilities: MidState Medical Center (MMC), a 156-bed acute-care hospital located in Meriden, CT in the central region and St. Vincent’s Medical Center (SVMC), a 473-bed community teaching hospital located in Bridgeport, CT in the southern region.

This article will be presented as a poster at the National Association of Orthopaedic Nurses (NAON) 41st Virtual Annual Congress on May 22, 2021.

Connecticut Orthopaedic Institute Pivot Plan

In line with directives from The Centers for Disease Control and Prevention and the United States Surgeon General, the COI’s parent healthcare system, HHC, postponed all elective surgeries starting March 19, 2020. In the initial wave of the pandemic, the peak incidence of COVID-19 occurred in Connecticut around April 22, 2020 [5-6]. The COVID-19 “flattening of the curve” was characterized by a sustained reduction in the rate of new COVID-19 cases for at least 14 days past the peak prevalence. At this point, healthcare leadership began to plan an effective and safe protocol to reintroduce orthopaedic surgeries and reopen the inpatient units. Beginning with a limited and carefully selected number of “time-sensitive and essential” orthopaedic procedures, we resumed elective surgeries on May 11, 2020 at MMC and on May 18, 2020 at SVMC. Following system-wide guidance for the resumption of care in operating rooms and in all procedural areas, the COI Pivot Plan revolved around four domains: safety, space, staff, and supplies. The plan was implemented across three phases of surgical care: Phase I: preoperative care, Phase II: inpatient care, and Phase III: postoperative discharge care. Challenges related to the four domains were as follows:

Safety

Safety was the preeminent consideration, encompassing all other Pivot Plan elements. COVID-19 is associated with a host of adverse postoperative outcomes in orthopaedic populations, as well as others. Thus, it is critical to ensure patient safety throughout the hospital stay [7-9]. Challenges under this domain included the need to minimize patient and staff exposure to COVID-19 through an array of preventive measures described below. Surgical-specific challenges associated with safety included the allotment of surgical time through a phased-gait process based on the degree of surgical complexity, which was updated and revised weekly.

Space

Challenges that were associated with this domain included reestablishing specialty care units and/or spaces that had been used to care for COVID-19 cases during the peak of the pandemic and ensuring that all geographical spaces in the hospital were adjusted and/or modified to provide the maximum “COVID-safe” environment. Additional challenges included the need to direct and control traffic to minimize people's interactions and equipment within specific spaces, as well as the need to accommodate a backlog of postponed elective surgeries and anticipated essential surgeries that would place additional demands on limited space.

Staff

During the peak of the pandemic, many surgical staff members had been re-deployed to various departments with the COVID-19 pandemic effort. In order to support the resumption of surgical procedures, the COI needed to recall these staff members to their original roles. Management had to carefully consider whether restaffing levels could support the resumption of surgical cases. Additionally, they had to decide if the units caring for COVID-19 patients could maintain adequate staffing levels without the continued support of the perioperative staff.

Supplies

Administrators and leadership needed to closely monitor the use and availability of all supplies, including personal protective equipment (PPE), medical/surgical supplies, medications, anesthesia supplies, and sterilization supplies.

## Materials and methods

In April 2020, the COI formed a multidisciplinary team of leaders that met weekly to design a Pivot Plan and an outcomes dashboard to guide the resumption of elective surgeries. Metrics were tracked weekly and were abstracted from Cipher Health, Premier, and Epic reports. Monthly metrics from May to November 2020 were compared to the six-month averages for the pre-pandemic baseline period September 2019 to February 2020. This project was reviewed by the Hartford Healthcare Institutional Review Board (Assurance #FWA00021932) and deemed to be exempt from IRB oversight as it did not meet the federal definition of research.

**Figure 1:**
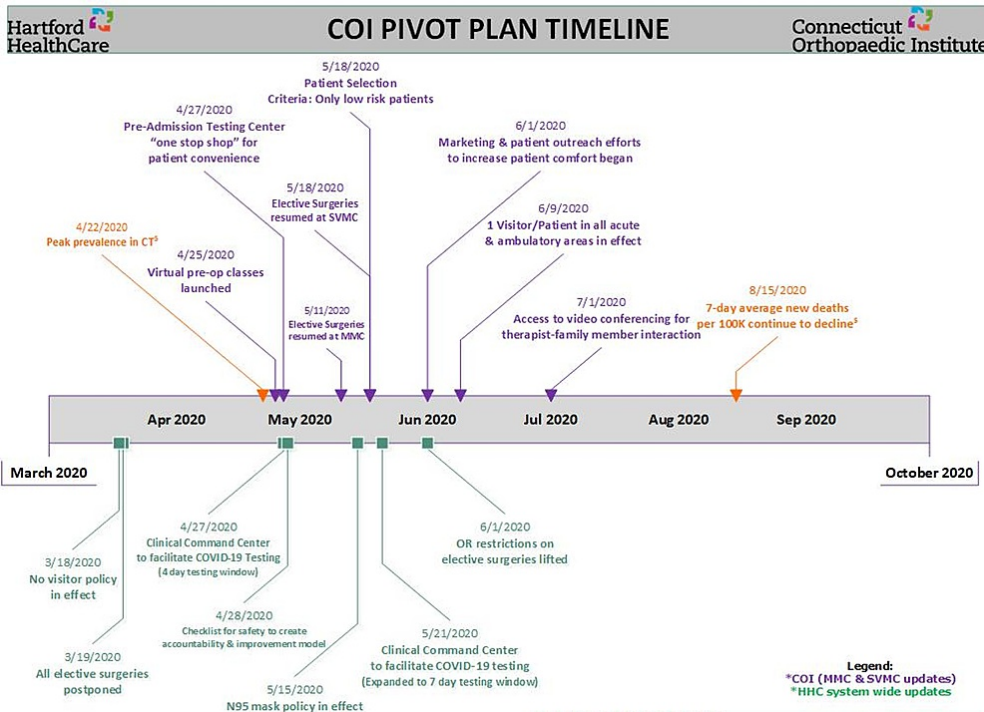
COI COVID-19 Pivot Plan timeline COI: Connecticut Orthopaedic Institute; CT: Connecticut; HHC: Hartford HealthCare; MMC: MidState Medical Center; OR: operating room; SVMC: St. Vincent’s Medical Center [5] CDC COVID Data Tracker. http://covid.cdc.gov/covid-data-tracker/#compare-trends_newcases (Accessed November 10, 2020)

The Pivot Plan elements implemented across the three phases are summarized below. Timelines for the implementation of these elements based on the localized effect of the pandemic at both sites are presented in Figure 1.

Phase I: preoperative care

Virtual Preoperative Education

Designed to minimize patient exposure to COVID-19, virtual preoperative education was initiated in March 2020 at SVMC and in April 2020 at MMC. A virtual online class was started at MMC, while SVMC began to offer a navigator-led class via telephone. As part of the virtual education, patients gained online access to educational materials available anytime from the convenience of their homes. MMC also began to offer live virtual webinars in addition to the existing online preoperative classes. Implementation of virtual preoperative education coincided with other system-wide efforts to reduce patient contact with COVID-19.

Clinical Command Center

The Clinical Command Center was implemented in April 2020 to facilitate the scheduling of preoperative COVID-19 testing as per HHC System Guidelines (Table 1). Along with the implementation of the Clinical Command Center, HHC developed a system-wide COVID-19 “dashboard” in the electronic health record (EHR) platform to allow providers to quickly and easily access the results of COVID-19 testing. Patients who tested positive were advised to cancel the surgery and were referred to their primary care physician (PCP) for further treatment. These patients could be retested as early as two weeks after the initial positive test, provided they were asymptomatic. Two negative tests, 24 hours apart, were required to reschedule the surgery. Testing was considered valid for staged/multiple procedures for seven days beyond the first procedure or up to a maximum of 14 days beyond the test date. On October 29, 2020, there was a system-wide update with regards to the COVID-19 retesting policy. All patients who tested positive within the previous three months did not need retesting as long as they were asymptomatic for at least 10 days after a positive test result.

**Table 1:** COVID-19 Screening and Testing for Surgical Patients PAC: Preadmission Testing Center; PCR: polymerase chain reaction

	Element
Screening	Uniform COVID-19 symptom and exposure screening questionnaires, including temperature checks, are performed at the following patient encounters: during the in-office appointment, In-person or via telehealth, during the preoperative history and physical, on the night before surgery, and on the day of surgery
Testing	A PCR test is to be performed within five days of the procedure starting April 27, 2020 (the window was expanded to seven days on May 21, 2020). A PCR test is ordered by the PAC or the designated care provider for the site. All surgical cases are reviewed for appropriateness as far in advance of the scheduled procedure as possible. The patient agrees to quarantine from the time of the test to the day of surgery. If the patient were to develop symptoms during that time, the surgery will be postponed.

Preadmission Testing Center (PAC)

The PAC was established as the initial patient contact after the patient was scheduled for surgery. The primary goals were to 1) risk-stratify patients preoperatively and facilitate consults as indicated, 2) call patients one to two weeks before surgery to facilitate medical clearance by offering history and physical (H&P) and preadmission testing, 3) address any last-minute test-related questions or concerns and reinforce hospital safety measures, 4) offer COVID-19 polymerase chain reaction (PCR) testing within seven days of the date of surgery (Table 1), 5) ensure patients remain in self-quarantine in-between the COVID-19 test and the procedure date, and 6) alert patients to any potential physical findings on the day of surgery which may lead to postponement or cancellation of surgery. MMC established a PAC in 2018 and they continued to offer services throughout the pandemic. SVMC expedited the launch of its PAC in April 2020 to accommodate patient demand for services due to the unavailability of primary care services as a result of the pandemic.

Patient Frequently Asked Questions (FAQs)

As scheduling resumed for limited orthopaedic procedures, the hospital and physician staff members received a list of FAQs in order to address their patient’s concerns regarding COVID-19 testing, safety, and preparation for surgery.

Risk Assessment Evaluations

Upon resumption of elective orthopaedic surgeries, orthopaedic navigators at MMC and SVMC performed risk assessment evaluations according to system-wide criteria. The criteria and rollout dates are presented in Table 2. If a patient was deemed high-risk, the surgeon was alerted and strongly encouraged to postpone the surgery. By mid-June, most restrictions on elective surgeries were lifted system-wide at which point weekly assessments were discontinued.

**Table 2:** Patient Selection Criteria for Roll-out of Elective Orthopedic Surgeries ASA: American Society of Anesthesiologists; BMI: body mass index; CPAP: continuous positive airway pressure; CRP: C-reactive protein; ESR: erythrocyte sedimentation rate; OSA: obstructive sleep apnea; RAPT: Risk Assessment Prediction Tool; RCRI: Revised Cardiac Risk Index

Risk stratification	Roll-out dates	Criteria
Low	MMC: Week of 5/11/2020 through 5/25/2020; SVMC: Week of 5/18/2020 through 6/1/2020	Age ≤ 75 years; ASA score 1 or 2; BMI < 40; metabolic level > 4; RAPT score > 10; STOP-Bang score < 5 (CPAP-compliant, OSA may be acceptable); RCRI < 2; Audit-C score < 2. No history of tobacco or nicotine use in last six months. No anticoagulant therapy for the last six months
High	MMC: 5/26/2020	Age > 75 years; extra caution for patients between the age of 65 and 75 years. History of chronic lung disease; history of heart disease; history/current diabetes mellitus; immunocompromised state; abnormal ESR and/or CRP; lymphopenia or neutropenia; Charlson Score ≥ 2; ASA score > 2; preoperative hemoglobin < 12 g/dL; RAPT score ≤ 9; STOP-Bang score > 4

Marketing and Patient Outreach Efforts to Increase Patient Comfort

Beginning June 2020, the COI 1) mailed flyers containing hospital safety data to patients who had not rescheduled elective surgeries, 2) followed up with patients via patient navigators, 3) posted recent patient testimonials about their experiences during the pandemic on the COI website, 4) created a 30-second advertisement addressing COVID-19 safety and the COI’s commitment to delivering a high level of care to our patients, 5) went live with providers on social media platforms and gave interviews on a local news station to reiterate COVID-19 safety, and 6) developed a safety message video for social media highlighting some of the COI amenities, as well as COVID-19 precautions.

Phase II: inpatient care

Preoperative Elements

With the return of elective orthopaedic surgeries, patients were required to wear masks to the hospital and began to be temperature and symptom screened at the front entrance (e.g., fever, cough, shortness of breath, gastrointestinal upset, etc.). The preoperative team reviewed COVID-19 test results and documented the patient’s and/or healthcare proxy’s desire to proceed with surgery amidst the COVID-19 pandemic. Other system-wide COVID-19 practices included limiting elevator capacity to a maximum of two persons at any one time, changing visitor restrictions (i.e., the zero visitor policy was edited to allow one visitor in June 2020), and changing the dress code for all staff (i.e., increased PPE guidelines with hospital-issued scrubs). 

Intraoperative Elements

In order to support orthopaedic surgeries that were deemed time-sensitive and essential for COVID-19-positive patients or a “person under investigation” (PUI), leadership at MMC and SVMC established a dedicated COVID-19 operating room (OR), which was decommissioned on June 29, 2020. With the return to elective surgeries, all staff was required to wear an N95 respirator in the operating room (OR). This requirement changed in July 2020 and was no longer required for patients with a documented negative COVID-19 test. All staff were required to wear and maintain their own surgical helmet and masks. As described under “Safety,” traffic through the OR was minimized under the surgeon’s direction.

Postoperative Recovery Elements

The Pivot Plan included the use of staggered patient bays in the post-anesthesia care unit (PACU) if sufficient room was available, and if bays held two patients, the curtain was utilized to separate the patients. Six-foot social distancing was implemented throughout the care process. Same-day (SD) surgical patients recovered and were transitioned to home from recovery spaces. Environmental Services increased the frequency of cleaning public spaces, lounges, and locker rooms.

Inpatient Elements

Infection prevention was key to navigating the “new normal.” HHC rolled out a system-wide checklist for safety on April 28, 2020 to create an accountability and improvement model for implementing, scaling, and sustaining best practices for infection prevention. Checklist elements included PPE and infection prevention, facilities/infrastructure, team member management, patient monitoring and screening, visitor monitoring and screening, performance management, and quality control. Additionally, HHC implemented an infection prevention audit tool to document general observations on cleaning, hand hygiene, appropriate social distancing, universal face masking, and PPE compliance. On August 31, 2020, HHC expanded its PPE guidelines as shown in Table 3 to help mitigate the possible spread of COVID-19 in clinical settings. A “No Visitor” policy was in effect system-wide as of March 18, 2020. Visitor restrictions were relaxed on June 1, 2020, but visiting hours continued to be limited. All patients were strongly advised to wear a mask at all times unless there was an underlying medical condition or other emergency prohibiting the use of a mask. In July 2020, videoconferencing was made available to enable therapist-family member interaction to provide emotional support and for physician-patient rounding. In May 2020, total joint group physical therapy was reduced from four patients to three. 

**Table 3:** System-wide PPE Guidelines AGP: aerosol generating procedure; PUI: patient under investigation

Patient status	PPE requirement
Non-COVID	Face shield or goggles + surgical mask
PUI/COVID+	Face shield + N95 + gown + gloves
AGP (Non-COVID/Non-PUI)	Face shield + surgical mask
AGP (PUI/COVID+)	Face shield + N95 + gown + gloves

Phase III: postoperative discharge care

In the postoperative phase, discharge transportation was arranged to observe social distancing rules. Post-discharge instructions regarding COVID-19 began to populate the discharge summary automatically. Increased touch-points between the navigator and patients were used to mitigate any complications, questions, or concerns that may occur postoperatively. Discharge planning staff began to receive weekly updates on the operation and percentage of COVID-19-positive patients at all local skilled nursing facilities.

## Results

Operational metrics are presented in Table 4. 

**Table 4:** Operational Metrics Note: SVMC resumed surgeries on May 18, 2020. With the smaller caseload, it was not necessary to use the same OR for back-to-back surgeries; therefore, turn-around time for that period was not applicable MMC: MidState Medical Center; OR: operating room; PACU: post-anesthesia care unit; SVMC: St. Vincent’s Medical Center

Metric	Baseline Sep 2019 - Feb 2020	May 2020	June 2020	July 2020	August 2020	September 2020	October 2020	November 2020
MMC								
Total case load	372	295	415	383	331	351	396	359
Average OR turn-around time (minutes)	42	43	40	42	38	39	37	38
Total PACU holds	12	19	2	11	5	7	9	9
Delays due to missing COVID-19 test results	0	0	0	0	0	0	0	0
SVMC								
Total case load	197	92	251	274	254	264	293	272
Average OR turn-around time (minutes)	50	N/A	30	46	50	52	47	52
Total PACU holds	15	5	19	17	20	33	2	18
Delays due to missing COVID-19 test results	0	0	0	0	0	0	0	0

The total caseload and the OR turn-around times were comparable pre and post-implementation of the Pivot Plan. The total number (N) of elective orthopaedic cases, including total joint arthroplasty (TJA), spine and podiatry cases in the six months prior to the coronavirus pandemic averaged at N = 372 cases per month for MMC and N = 197 for SVMC, respectively. Since the resumption of elective surgeries in May 2020, the average total elective orthopaedic caseload per month was N = 361 (high 415, low 295) for MMC and N = 243 (high 293, low 92) for SVMC. PACU holds (which occur when patients are held in the PACU for non-medical reasons) have been decreasing over time at MMC (corresponding to the need to maintain social distancing). While PACU holds were increasing at SVMC, they remained close to the pre-COVID baseline. Neither site experienced delays in surgeries due to missing COVID-19 test results. When elective surgeries resumed in May, more than 50% of COI patients used the PAC as a “one-stop-shop” for history and physical, pre-admission testing, as well as COVID-19 testing. Between March 2020 and May 2020, 489 cases were canceled at MMC, and 126 cases were canceled at SVMC due to the pandemic. By the end of October 2020, 83% and 90% of canceled cases at SVMC and MMC, respectively, had been rescheduled.

Quality metrics are presented in Table 5. 

**Table 5:** Quality Metrics Note: SVMC joined the COI in September 2019. SVMC uses a different platform to track postoperative navigator calls, and therefore, we only have access to data on patients needing a postoperative follow-up call from MMC. DVT: deep vein thrombosis; MMC: MidState Medical Center; PE: pulmonary embolism; SVMC: St. Vincent’s Medical Center

Metric	Baseline Sep 2019 - Feb 2020	May 2020	June 2020	July 2020	August 2020	September 2020	October 2020	November 2020
MMC								
% of patients requiring follow-up call after surgery	25.0	23.0	24.0	24.0	27.0	30.0	36.0	30.0
% of patients readmitted within 30 days of surgery	1.43	1.85	1.06	1.35	1.40	0.67	1.19	0.58
% of patients discharged on the same day	8.0	27.0	15.0	16.0	11.0	12.0	14.0	22.0
% of patients with COVID-19 diagnosis within 23 days after surgery	0	0	0	0	0	0	0	0
% of patients with postoperative DVT/PE	0.0	0.0	0.0	0.0	0.0	0	0	0
% of patients transitioned to home	96.8	100	98	97	95	99	98	99
SVMC								
% of patients requiring follow-up call after surgery	-	-	-	-	-	-	-	-
% of patients readmitted within 30 days of surgery	2.73	0.0	0.0	3.0	5.0	0.0	0.0	0.0
% of patients discharged on the same day	3.0	11.0	13.0	6.0	11.0	15.0	13.0	11.0
% of patients with COVID-19 diagnosis within 23 days after surgery	0.0	0.0	0.0	0.0	0.0	0.0	2.0	0.0
% patients with postoperative DVT/PE	0.0	0.0	0.0	0.0	0.0	0	0	0
% patients transitioned to home	88	100	98	97	98	98	97	95

SD discharge rates for total hips and knee replacements in the six months prior to the pandemic averaged 8% for MMC and 3% for SVMC. From May to November 2020, the average was 16.7% (high 27%, low 11%) for MMC and 11.4% for SVMC (high 15%, low 6%). Additionally, the 30-day readmission rate for hip and knee replacements was also recorded, with the six-month pre-COVID average being 1.4% for MMC and 2.7% for SVMC. After the seven-month data collection, the average for MMC and SVMC was 1.1%. The six-month baseline average for transitions to home (T) from the inpatient setting was 96.8% for MMC and 88% for SVMC. Data recorded during the pandemic suggested an average of 98% (high 100%, low 95%) for MMC and an average of 97.6% (high 100%, low 95%) for SVMC. No patients developed deep vein thrombosis (DVT) or pulmonary embolism (PE) postoperatively during the study period. Lastly, all TJA patients were contacted 23 days after discharge to assess for positive COVID-19 diagnosis. After seven months of data collection, the rate was at 0% at MMC and 0.2% at SVMC. 

## Discussion

**Figure 2:**
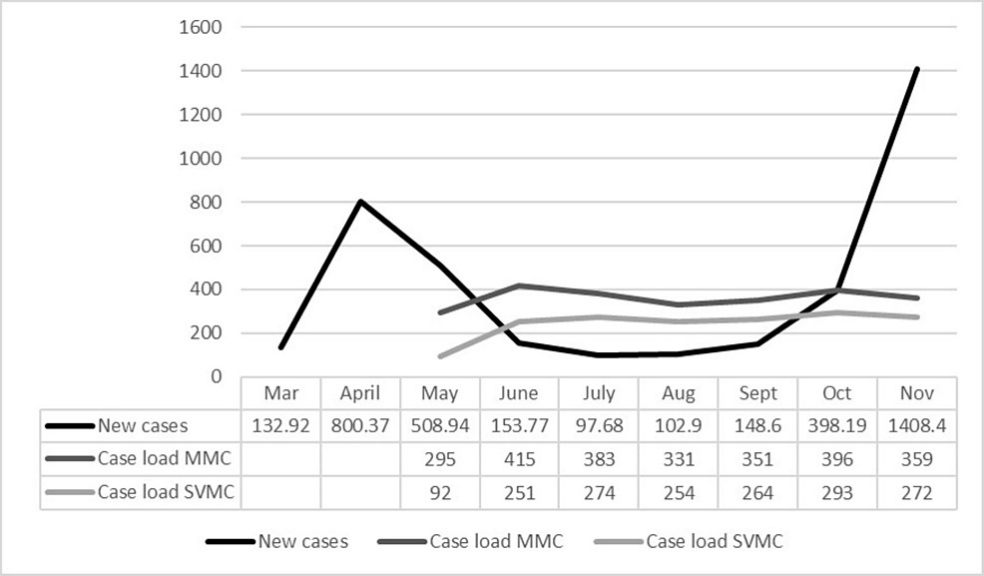
New cases of COVID-19 and total caseloads for COI The graph depicts the monthly average of seven-day rolling averages of new COVID-19 cases in Connecticut and the total case load per month for COI at MMC and SVMC. Data for March 2020 represents March 19 – March 31, 2020. COI: Connecticut Orthopaedic Institute; MMC: MidState Medical Center; SVMC: St. Vincent’s Medical Center Source: CDC COVID Data Tracker (http://covid.cdc.gov/covid-data-tracker/#compare-trends_newcases) and COI Pivot Plan dashboard [5]

Healthcare systems faced with reinstating elective arthroplasty in the midst of the ongoing pandemic must balance patients’ demand for these procedures with the concomitant need to mitigate the risk of exposure to COVID-19. As systems postponed elective surgeries, patients awaiting hip and knee arthroplasty were faced with waiting periods of unknown duration characterized by extreme discomfort and severely impaired quality of life [10-11]. Patient safety is key to the resumption of elective orthopaedic surgery as COVID-19 presents an additional risk of morbidity and mortality beyond the regular risks already associated with these procedures [10]. Compounding the situation and further underscoring the need to identify a safe plan for the resumption of surgery is the additional risk of opioid, alcohol, and other substance use disorders in patients faced with often debilitating pain [11]. Our dashboard metrics support the success of the COI Pivot Plan in terms of both efficiency and patient safety. Elective surgeries resumed at MMC on May 11, 2020, and by June, they recorded 415 elective orthopaedic surgical cases, marking the highest monthly volume on record since the inception of the institute in 2017. As SVMC resumed elective surgeries on May 18, 2020, 251 elective orthopaedic surgical cases were completed in June compared to the pre-COVID six-month baseline average of 197 cases. In addition, despite the surge of new COVID-19 cases that began to spike again in September 2020, the COI caseload remained consistent as shown in Figure 2. This illustrates the effectiveness of the Pivot Plan in allowing the COI to continue to maintain operations in the midst of the worsening pandemic. These numbers, when compared to pre-COVID data, indicate that the COI did not experience a loss of elective surgical volume due to the coronavirus pandemic. Previous work has indicated that patients experiencing hip and knee arthritis are still eager to have surgery, even in light of the current pandemic [12]. The fact that the COI rescheduled 80% - 90% of its canceled surgeries illustrates the patient demand for a safe and effective return to orthopaedic procedures on the local level. It is important to note that it was through our marketing and patient outreach efforts that we were able to effectively communicate all the COVID-19 precautions to instill confidence in our patients that we were ready to provide safe care across the continuum of care.

Data on SD discharges indicate that orthopaedic providers were cognizant of the length of stay and were diligent with efficient discharge after clinical criteria were met in order to reduce the risk of in-hospital exposure to COVID-19. Historically, approximately 8% of patients undergoing lower extremity TJA were discharged on the same day at MMC. With the implementation of additional precautions and return to elective surgery, this number climbed to 27% in May 2020, likely due to patient and provider fear of viral exposure during a prolonged hospital stay. It is worth noting that these initial patients were scheduled based on overall health conditions and limited comorbidities. The program’s nurse navigators risk-stratified these patients according to predetermined criteria that deemed them low-risk. However, in the seven months following the resumption of surgery, the SD discharge rates remained elevated. No month was recorded at or less than the baseline of 8% at MMC. In the month of August 2020, the program at MMC recorded the lowest rate of 11%, which was still higher than pre-COVID rates. In fact, the average rate of SD discharge throughout this time was 16.7%. A similar upward trend was observed at SVMC with an average rate of SD discharge of 11.4% compared to the pre-COVID six-month average baseline of 3%. This can perhaps be attributed to COVID-19 positivity rates at their lowest in the state, in addition to training the staff for SD discharge without any lapse in the care needed postoperatively as part of the Pivot Plan [5].

Although SD discharge rates were more than double relative to baseline, the data illustrate that we maintained a high level of care, as reflected in the comparable rates of 30-day readmissions (30R) pre- and post-implementation of the Pivot Plan. Further, no patients experienced deep vein thrombosis (DVT) or pulmonary embolism (PE), despite the fact that previous groups have reported an increased incidence of venous thromboembolism (VTE) in patients undergoing elective arthroplasty during the pandemic, possibly due to the need to self-quarantine for a prolonged time before surgery [13]. In six out of seven months studied, we noted that the 30R was lower than the pre-COVID average, with the lowest recorded rate of 0.5% in the month of November 2020 at MMC and 0% in the months of May, June, September, October, and November 2020 at SVMC. These data suggest that the quality of patient care had improved. The coronavirus may have limited the patient’s willingness to return to the emergency department. However, patients could alternatively be evaluated in their surgeon's private office and could be directly admitted to the institute’s inpatient floor for further care. Only the month of May 2020 recorded a higher-than-normal 30R of 1.8% at MMC. This finding is likely multifactorial, although it is probable that a seven-week surgical hiatus played a consequential role.

Transitions for TJA patients to their home settings after surgery also reflect the quality of care and the efficiency of the patient throughput process with necessary precautions in place. Prior to the severe acute respiratory syndrome coronavirus 2 (SARS-CoV-2) pandemic, the percentage of patients discharged home from MMC was 96.8%. During the pandemic, about 98% of patients were discharged home, indicating that the pandemic had no effect on discharge planning and placement at MMC. However, at SVMC, we observed a pronounced effect on patients transitioning to home with 97.6% compared to 88% pre-COVID, likely due to patient inclination for discharge to home, as well as the localized effect of the pandemic in the southern region that is in close proximity to New York City.

Perhaps most illustrative of the successful implementation of precautions were the rates of postoperative COVID-19 diagnoses after hospital discharge. This information was reported by the patients during the postoperative telephone consult 23 days after patient discharge. With the resumption of elective orthopaedic surgeries in May, the COVID-19 diagnosis was 0% at MMC and 0.2% at SVMC, demonstrating the effectiveness of the precautions put in place at the system level. All patients were pre-screened with a COVID-19 PCR nasopharyngeal swab within four days of their procedure and then asked to quarantine up to the day of surgery. Additional precautions, such as a virtual preoperative class and a centralized testing center, helped patients reduce their exposure to the virus. In addition, an update to the history and physical note on the day of surgery needed a confirmed negative test result for COVID-19 in order to continue with the planned procedure. All surgical staff were also screened upon entry to the hospital with temperature checks and a COVID-19 questionnaire. Surgeries were rescheduled when patients experienced symptoms seven days prior to their procedure or had a positive PCR swab.

Many elements of the COI Pivot Plan have also been incorporated by other institutions faced with restarting non-emergent arthroplasty during the ongoing pandemic. Screening and testing for COVID-19 prior to surgery, streamlining the presurgical process by limiting in-person contact, stringently utilizing PPE, practicing social distancing, prioritizing patients based on COVID-19 risk, and adapting the pre- and perioperative environment to limit the risk of infection are some of the elements shared in common with plans put forth by other institutions [14-15]. The present report adds to the evidence base of sound practices in proceeding with elective surgeries during a pandemic and could inform the efforts of other institutions seeking to restart non-emergent surgeries during similarly trying times in the future. 

## Conclusions

Through the collaboration of a multidisciplinary team, the COI Pivot Plan was successfully implemented at two different hospitals offering elective orthopaedic surgeries to a varied patient population. Data tracked through the dashboard support the effectiveness of the Pivot Plan in allowing the COI to safely resume elective orthopaedic surgeries. The precautions taken by COI in terms of safety, space, staff, and supplies across the preoperative, inpatient, and postoperative/discharge phases of care were effective in controlling the spread of the SARS-CoV-2 virus after the reinstatement of elective orthopaedic surgery. Furthermore, data collected before and after the onset of the COVID-19 pandemic indicated that the COI either maintained or improved upon key operational and quality measures. Moving forward, continued flexibility of staff, administration, and the healthcare system as a whole, as well as transparency between healthcare providers and patients, will be key to its continued success. 
